# A Procedure for Oxytocin Measurement in Hair of Pig: Analytical Validation and a Pilot Application

**DOI:** 10.3390/biology10060527

**Published:** 2021-06-13

**Authors:** Marina López-Arjona, Fernando Tecles, Sandra V. Mateo, María Dolores Contreras-Aguilar, Silvia Martínez-Miró, José Joaquín Cerón, Silvia Martínez-Subiela

**Affiliations:** 1 Interdisciplinary Laboratory of Clinical Analysis of the University of Murcia (Interlab-UMU), Regional Campus of International Excellence ‘Campus Mare Nostrum’, University of Murcia, Campus de Espinardo s/n, 30100 Espinardo, Murcia, Spain; marina.lopez10@um.es (M.L.-A.); ftecles@um.es (F.T.); sandra.valverde@um.es (S.V.M.); mariadolores.contreras@hotmail.com (M.D.C.-A.); silviams@um.es (S.M.-S.); 2 Departament of Animal Production, Regional Campus of International Excellence ‘Campus Mare Nostrum’, University of Murcia, Campus de Espinardo s/n, 30100 Espinardo, Murcia, Spain; silviamm@um.es

**Keywords:** oxytocin, hair, pig, methanol

## Abstract

**Simple Summary:**

Oxytocin is gaining importance in human and animal studies because of its role in welfare stress response, but there is no evidence of oxytocin measurement in hair, which would allow an evaluation of this analyte over a long period of time. For this reason, in this study, a new immunoassay to measure oxytocin in the hair of pigs was developed and validated. In addition, the possible changes in concentrations of oxytocin in hair during the reproductive cycle of pigs were evaluated. The assay was precise and accurate and when applied to the hair extracts of pigs, higher oxytocin values were obtained at days 23 and 59 after farrowing in the winter–spring period. When oxytocin concentrations were compared to cortisone and cortisol, it showed moderate and low correlations, respectively. Based on the results of this report, oxytocin can be measured in the hair of pigs, and changes in concentrations can be detected during a pig’s reproductive cycle.

**Abstract:**

There is growing interest in oxytocin as a biomarker of stress and welfare. The objective of this study was to develop and validate a procedure based on a highly sensitive immunoassay to measure oxytocin in the hair of pigs. In addition, a pilot study to apply this procedure to evaluate possible changes in concentrations of oxytocin in hair during the reproductive cycle of pigs at different periods of the year was conducted. This procedure used methanol for sample extraction, since it offered better recoveries than acetonitrile, and the immunoassay developed was precise and accurate for the quantification of the oxytocin in the hair. When this procedure was applied to hair collected at different times of the reproductive cycle and season, higher values were found at days 23 and 59 after farrowing in the winter–spring period. In addition, higher oxytocin values in the spring–summer period were found in hair collected 5 days before farrowing compared to winter–spring. Oxytocin in hair showed moderate and low correlations with cortisone and cortisol in hair, respectively. This study represents the first report in which oxytocin was measured in hair and could open new lines for future research about the measurement of oxytocin in pigs and other biological species as a biomarker of stress.

## 1. Introduction

Oxytocin is related to social recognition and attachment in mammals [1]. Over the last years, it has gained high interest as a biomarker for welfare evaluation in farm animals [2]. In the pig, particularly, salivary oxytocin has been used to study human–animal interaction [3], and there have been described increases in this analyte in saliva in farrowing [4] or after ejaculation [5].

Hair has been used as a non-invasive way to evaluate the stress response in animals through the measurement of cortisol [6] and cortisone [7]. In addition to the advantage of being a non-invasive sample, in some cases, such as cortisol, hair is useful in the evaluation of the long-term evolution of the dynamics of this analyte in the body [7,8,9,10]. In pigs, previous reports indicated that hair samples can provide information to a period varying from one to two months before the date of collection [8]. However, to the authors’ knowledge, there are no studies about the oxytocin measurement in hair.

The hypothesis of this study was that oxytocin could be measured in hair samples of pigs. To test the hypothesis, the objective of this manuscript was to develop and validate a procedure to measure oxytocin in the hair of pigs based on a highly sensitive immunoassay. In addition, a pilot study was conducted in which changes in oxytocin concentrations in hair were evaluated during the reproductive cycle of pigs at different periods over the year.

## 2. Materials and Methods

### 2.1. Oxytocin Assay

For the measurements of oxytocin, an amplified luminescent proximity homogenous assay that uses two bead types (donor and acceptor beads) was used, based on the AlphaLISA technology (PerkinElmer Inc., Waltham, MA, USA). The assay used in this study was a direct competitive format, with a monoclonal antibody against oxytocin obtained previously [4] that does not have cross-reactivity with vasopressin [4]. The assay was performed in 96-well plates (PerkinElmer Inc., Waltham, MA, USA) with a total volume of 50 µL per well. For assay optimization, varying concentrations of biotinylated oxytocin (0–3 nM), monoclonal anti-oxytocin antibody coated to acceptor beads (20–40 µg/mL) and donor beads (PerkinElmer Inc., Waltham, MA, USA) (20–40 µg/mL) were evaluated.

### 2.2. Selection of the Solvent for the Sample Extraction Procedure

In the first stage, to determine which solvent would be better for the extraction of oxytocin from hair, two different chemicals were tested. One was methanol, which has been demonstrated to be efficient for cortisol and cortisone extraction [7,8,9,10,11], and the second was acetonitrile, which has been used previously for oxytocin extraction from urine [12] or saliva [3].

In both cases, the procedure for hair processing was the same, and it was based on a previous procedure used for cortisol [13] and cortisone [7] extraction. The hair samples from the sows were weighed (250 mg), washed with isopropanol twice, and left at room temperature (RT) until completely dry. The hair samples were cut into small pieces and 60 mg from each sample was weighed and pulverized into a fine powder in a homogenizer (Precellys Evolution homogenizer, Bertin Technologies, Montigny-le-Bretonneux, France). The pulverized hair was incubated with 1 mL of the solvent for 18 h at RT with continuous gentle agitation for oxytocin extraction. Samples were centrifuged and 0.6 mL of the extract was aliquoted into a new Eppendorf tube. The samples were evaporated to dryness in a Speed Vac Concentrator (Concentrator 5301, Eppendorf, Hamburg, Germany). The dry extracts were reconstituted with 0.1 mL of phosphate buffer saline (PBS) and analyzed.

The evaluation of the efficiency of two different solvents (i.e., methanol and acetonitrile) for oxytocin extraction was made based on the procedure by Madry et al. (2018) [14]. A standard solution of purified oxytocin (Oxytocin-BSA, Cusabio, Houston, TX, USA) at a concentration of 1000 pg/mL was added to four different pools of hair samples after the pulverization. Then, 1 mL of extraction solvent (methanol or acetonitrile) was added. The processing method was the same for all of the samples, using the method described above. At the same time, the same four pools of hair samples were processed without the addition of the standard. Oxytocin was measured in all samples, and the results expressed as pg/mg of hair.

### 2.3. Assay Validation

Oxytocin standards were prepared by diluting conjugated oxytocin (Oxytocin–BSA) in AlphaLISA Universal buffer. The standard curve was generated using 8 standards at concentrations of 2400, 1200, 600, 300, 150, 75, 37.5, and 0 pg/mL. For analytical validation of the assay, imprecision was calculated as inter- and intra-assay variations and expressed as coefficients of variations (CVs). For the intra-assay precision of the method, five replicates of two pools (hair extracts with high and low oxytocin concentrations extracted with methanol, which in the previous experiment gave the best results) were analyzed at the same time. Five aliquots of each pool were stored in plastic vials at −80 °C until analysis. For inter-assay precision, these samples were measured in duplicate five times, over five different days using freshly prepared calibration curves.

The accuracy was evaluated by an assessment of linearity under dilution and recovery experiments. For the linearity evaluation, two hair extracts were serially diluted from 1:2 to 1:128 with AlphaLISA Universal buffer. For the recovery test, different concentrations (600, 300, 150, and 75 pg/mL) of conjugated oxytocin (Oxytocin–BSA) were added to the hair extracts with known oxytocin concentrations and the percentages of the measured to the expected oxytocin concentrations were calculated.

The limit of detection (LD) was obtained by the mean + two standard deviations (SDs) of 12 replicated measurements of the AlphaLISA Universal buffer. The limit of quantification (LLOQ) was calculated based on the lowest oxytocin concentrations that could be measured with less than 20% imprecision. For this, hair extracted samples with a known oxytocin concentration were serially diluted from 1:2 to 1:128 and analyzed five times at the same run for each dilution.

### 2.4. Study Animals and Sampling

Stored samples from a total of 20 multiparous Large White sows, which were used in a previous research to measure cortisol and cortisone in hair of pigs, were included in this study [7].

Briefly, all sows were housed at the Experimental Farm of the University of Murcia located in Guadalupe (Murcia, Spain). The sows had *ad libitum* access to water and were fed 2.5 kg of a commercial nutritionally balanced diet once daily (at 8:00 a.m.). Ten sows were sampled during one reproductive cycle and the other ten in a subsequent reproductive cycle, with individuals in each group sampled at 5 days before expected farrowing and at 23 and 59 days after farrowing. Of the 20 sows, 10 were sampled from April to September 2018 (spring–summer period: S–S) and the remaining 10 from January to June 2019 (winter–spring period: W–S). The RT where the sows were housed was recorded using data loggers at each time of sampling. At these sites, in the S–S period, a range of mean temperatures at different farrowing times from 24.8 to 25.7 °C were recorded, where the range in the W–S were from 18.6. to 22.4 °C.

Hair samples were collected between 9:00–10:30 a.m. The hair was cut manually using scissors, close to the skin on the rump region, and alternated between the right and left sides, avoiding any contaminated areas. The samples were placed in small plastic bags, individually marked and stored at RT until their processing. The research protocols were approved by the Bioethical Commission of Murcia University according to the European Council Directives regarding the protection of animals used for experimental purposes (Approval number: 235/2016; Approval date: 25 April 2016).

For oxytocin measurement in hair samples, the assay developed in this study was used. For the cortisol and cortisone determination, an indirect competitive alphaLISA was used in the previous study [7]. For cortisol determination, a commercially available monoclonal antibody against cortisol obtained from a mouse was used, while for cortisone determination, a polyclonal antibody against cortisone was used.

### 2.5. Statistical Analysis

Medians, SD, CVs, recovery efficiency, and linear regression analyses were calculated in a spreadsheet using routine descriptive statistical procedures (Excel 2019, Microsoft). The Shapiro–Wilk test was used to evaluate the normality of the distribution of data obtained from the different experiments, giving a nonparametric distribution, so the data was transformed logarithmically for evaluating the differences in oxytocin concentrations. Two-way repeated measures analysis of variance (ANOVA) and the uncorrected Fisher’s least significant difference (LSD) test for multiple comparisons were used to determine if there were any significant differences in the values obtained for oxytocin at different phases during the reproductive cycle and at different periods of the year. The strength of the Spearman correlation between oxytocin concentrations obtained in this study and cortisol, cortisone, and 11β-hydroxysteroid dehydrogenase (11β-HSD) type 2 activity previously measured [7] was assessed by the rule of thumb [15], according to which an *R* value between 0.90 and 1 was considered to have very high correlation, 0.70–0.90 to have high correlation, 0.50–0.70 to have moderate correlation, 0.30–0.50 to have low correlation, and less than 0.30 little, if any, correlation. These statistical analyses were calculated using GraphPad Prism 8 (GraphPad Software).

## 3. Results

### 3.1. Selection of the Solvent for the Extraction Procedure

The extraction recovery efficiency for each solvent is shown in Table 1. In case of extraction with methanol, the extraction efficiency average of the four samples was 103.4 ± 19.9, while in the case of acetonitrile, it was 57.3 ± 19.7.

### 3.2. Oxytocin Assay Validation

**Figure 1:**
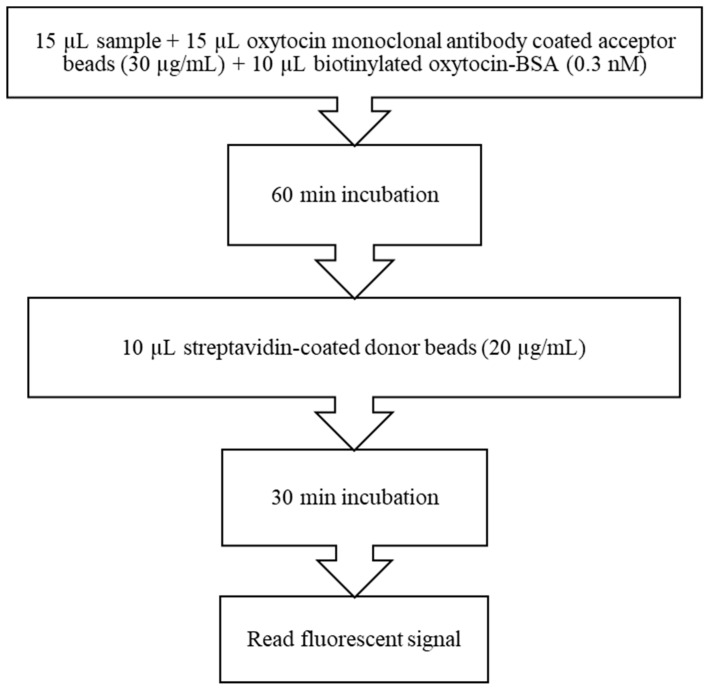
AlphaLISA protocol for hair oxytocin measurement in pigs.

The optimal conditions of the assay for oxytocin measurement in hair are shown in Figure 1.

The immunoassay for oxytocin measurement in hair showed an intra-assay imprecision of 3.8% and 2.6% for samples with high and low hair oxytocin concentrations, respectively, and an inter-assay imprecision of 8.3% and 9.5% for samples with high and low hair oxytocin concentrations. Dilution of hair samples from 1:2 to 1:64 resulted in linear regression equations with a correlation coefficient of 0.99. The average recovery obtained a range between 80% and 103%. The assay’s LD was 30.9 pg/mL, and the LLOQ was 0.2 pg/mg of hair.

### 3.3. Hair Oxytocin Concentrations in the Experimental Trial

The ANOVA analysis showed a significant effect on the phase of the reproductive cycle and period of the year (*p* = 0.0032).

**Figure 2:**
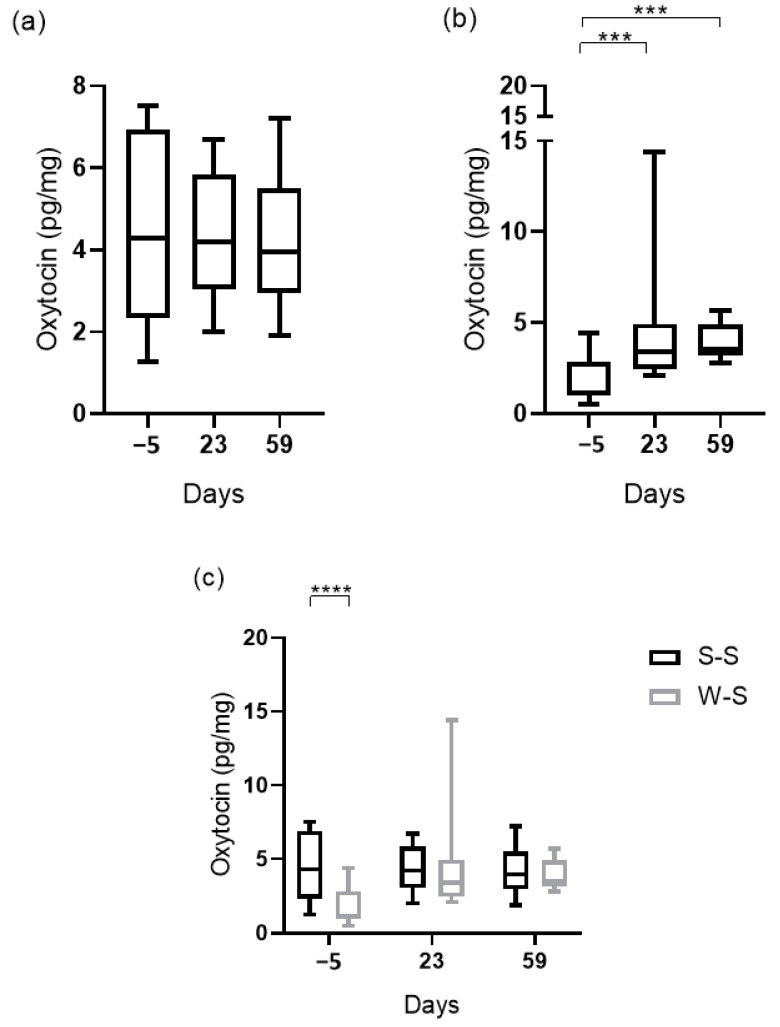
Oxytocin concentrations (pg/mg) in hair samples of sows at 5 days before farrowing (−5), 23 days after farrowing (23), and 59 days after farrowing (59) in spring–summer (*n* = 10) (**a**); winter–spring (*n* = 10) (**b**); and in both periods (S–S, spring–summer; W–S, winter–spring) on the same sampling day per period (**c**). Asterisks indicate significant differences (**** *p* < 0.0001; *** *p* < 0.001). Plots show median (line within box), 25th and 75th percentiles (box) and minimum and maximum values (whiskers).

Oxytocin concentrations on different phases in the reproductive cycle and period of the year are shown in Figure 2.

In the S–S period, oxytocin in hair did not show significant differences among days of the reproductive cycle (*p* > 0.05), while in the W–S period, oxytocin concentrations were higher at day 23 after farrowing (median, 3.4 pg/mg; 25–75th percentile, 2.5–4.9 pg/mg; *p* = 0.0001) and day 59 after farrowing (median, 3.5 pg/mg; 25–75th percentile, 3.2–4.9 pg/mg; *p* = 0.0002) than at day 5 before farrowing (median, 1.1 pg/mg; 25–75th percentile, 0.9–2.8 pg/mg).

Oxytocin concentrations in hair showed significantly higher values at day 5 before farrowing in the S–S period compared with W–S (median, 4.3 pg/mg; 25–75th percentile, 2.3–6.9 pg/mg vs. median, 1.1 pg/mg; 25–75th percentile, 0.9–2.8 pg/mg; *p* < 0.0001). Although the values of oxytocin tended to be higher in all sampling times obtained in the S–S period, there were no significant changes depending on the season at day 23 or 59 (*p* > 0.05).

### 3.4. Correlation of Oxytocin with Cortisol, Cortisone, and 11β-HSD Type 2 Activity

There was a significant moderate correlation of oxytocin concentrations with cortisone concentrations in hair (*R* = 0.663, *p* < 0.0001) and a significant low correlation with cortisol concentrations (*R* = 0.316, *p* = 0.008). In addition, the correlation with cortisone/cortisol (Cn/C) ratio (11β-HSD type 2 activity) was moderate (*R* = 0.509, *p* < 0.0001).

## 4. Discussion

This study reports the measurement of oxytocin concentrations in the hair of pigs. Previous studies have used hair samples for the measurement of selected biomarkers, such as cortisol or cortisone [7,8], but to the authors’ knowledge there are no studies about oxytocin in hair in any animal species or humans. The assay method developed showed high precision and accuracy as well as a low LD and LLOQ, being suitable for the measurement of oxytocin at the concentrations that appear in hair. This assay also has several advantages, such as the low volume of sample needed, lack of required washing steps, and a shorter processing time. Moreover, the use of hair over other sample types, such as blood and saliva, also has advantages including the ease of transport and storage.

In our experimental procedure, methanol produced a better oxytocin recovery than acetonitrile. It is important to point out that these results are only valid for our experimental conditions and specific assay and should be validated with other types of sample extraction or assays. Methanol has been used as a solvent for oxytocin with the advantage of not producing any conformational changes in this analyte [16] and have also been used for studies of oxytocin extraction from milk [17]. Although acetonitrile has been used for oxytocin extraction in some samples, such as saliva [3], previous studies do not recommend acetonitrile as extractor medium for selected drugs because of the low efficiency of extraction [14].

The interpretation of the results obtained in our experimental procedure should be taken with caution, since there is still lacking proper knowledge of the adequate interpretation of oxytocin results in hair. In general, higher values of oxytocin during the S–S period, which had higher external temperatures, were obtained. In this line, increases in oxytocin concentrations in the serum of ewes [18] after exposure to high temperatures have been described. Although the reason for the increase in oxytocin is not clear, in the case of the experiment with the ewes, it could be related to the secretion of vasopressin (a very similar peptide), which is made in cases of externally high temperatures to reduce water loss [18]. This should be considered a hypothesis, because in other species, such as rats [19], no changes were seen in oxytocin serum after thermal stimulation. Therefore, other factors, such as the number of hours of sunlight per day (although this was not controlled in our study) could influence the differences in oxytocin between S–S and W–S [20].

Regarding the evolution of oxytocin concentrations along the reproductive cycle of the sows, there was an increase after the farrowing and the lactation period in the W–S period. Interestingly, there was a moderate correlation between oxytocin and cortisone in hair, and increases in cortisone concentrations in these samples in the same period have been described [7]. These increases could be due to the increase in the activity of 11β-HSD type 2, which is the enzyme in charge of the cortisol to cortisone conversion, and it is expressed close to oxytocin in the hypothalamus [21]; therefore, there could be a relation between both analytes. Although oxytocin has been traditionally related to psychological and social well-being in domestic animals [22], increases in central and peripheral oxytocin concentrations have been found in response to some stressful stimuli in human, such as distressed pair–bond relationships [23] or anxiety [24], and also in animals under stress, such as shaker stress in rats. These increases could be a reaction by the organisms toward suppressing stress, improving the stress tolerance in farm animals [2], and could help to adapt to a stressor over time [25]. Along this line, oxytocin has, according to the allostatic theory, an inhibitory action on the activity of hypothalamic–pituitary–adrenal axis [26].

As limitations of the study, the method developed has not been compared with mass spectrometry, although the common mass spectrometers fail to detect different forms of oxytocin and not detect oxytocin bound to other molecules [27]. In addition, no specific stability studies have been done to evaluate if the oxytocin is stable after methanol extraction at −80 °C. Moreover, the interpretation of the changes found in our study was mostly speculative, since the relationship between oxytocin in hair and circulation is not known and, therefore, it cannot be stated which period of time for the oxytocin values in circulation would reflect a determined length of hair. This should be clarified in future research, with studies in which oxytocin in hair and blood are compared. These studies could follow the model of previous reports in which, for example, the possible relationship between cortisol in hair and saliva was studied [28]. In addition, it would be interesting to evaluate the possible different forms of oxytocin that could appear in hair, as in the case of saliva [29], and the response of oxytocin in hair to different situations of acute or chronic stress.

## 5. Conclusions

Oxytocin can be measured in the hair of pigs. Oxytocin concentration in hair can increase at farrowing or lactation as well as in high environmental temperatures. These data open a new field for studies about oxytocin and their possible applications as a biomarker of stress.

## Figures and Tables

**Table 1:** Oxytocin concentrations in the hair pool samples (1–4), concentrations of the internal standard, extraction efficiencies, and recovery averages (mean of the four ± each standard variation) of the extraction procedures with methanol and acetonitrile.

Sample	Extractor	Internal Standard	Internal Standard (pg/mL)	Sample Concentration (pg/mL)	Expected Value (pg/mL)	Obtained Value (pg/mL)	Extraction Efficiencies (%)	Recovery Average ± Standard Deviation (%)
Sample 1	Methanol	Oxytocin–BSA	1000	417.4	708.7	657.4	92.7	103.4 ± 19.9
Sample 2	Methanol	Oxytocin–BSA	1000	293.9	646.9	778.8	120.4	
Sample 3	Methanol	Oxytocin–BSA	1000	159.0	579.5	695.1	119.9	
Sample 4	Methanol	Oxytocin–BSA	1000	44.1	522.1	421.5	80.7	
Sample 1	Acetonitrile	Oxytocin–BSA	1000	370.0	685.0	520.6	76.0	57.3 ± 19.7
Sample 2	Acetonitrile	Oxytocin–BSA	1000	225.6	612.8	417.9	68.2	
Sample 3	Acetonitrile	Oxytocin–BSA	1000	42.3	521.2	163.2	31.3	
Sample 4	Acetonitrile	Oxytocin–BSA	1000	77.9	538.9	288.5	53.5	

## Data Availability

Not applicable.
